# Report on Therapeutics

**Published:** 1878-02

**Authors:** Frank M. Odell


### REPORT ON THERAPEUTICS.

*Read before the American Dental Association, Aug. 6, 1877.*

BY FRANK M. ODELL, D. D. S.

The term therapeutics comes from the Greek *therapcutikos*, curative, or that which pertains to the healing art; from *therapio*, to nurse, to serve to cure: From which we deduce that therapeutics is that part of medicine which treats of the way of curing disease; and, in its more extended signification, it treats of the symptoms, the conclusions to be drawn therefrom; the power of nature, and how far it may be relied upon for the correction of pathological condition, of the mode of treatment to be adopted, and of the different symptoms of practice which have acquired reputation. With some the term is also used to indicate the modus operandi by which the action of medicine is manifest.

Whilst prophylaxis would indicate the art of preserving health, therapeusis would be the art of restoring it when lost; or to speak more exactly, when dominated by pathological activities. According to this definition, therapeutics is a science of such boundless extent as to embrace all the collateral branches of the science and art of medicine. Therefore, whether treating of Iatrology, histology, biology, physiology, pathology, botany, materia medica, toxicology, or even of geology, mineralogy, conchology, ichthology, ornithology, or a host of other ologies, alogies and isms, to show that the former practices of physicians, or their classification of, or manner of employing remedies, that the histogenesis or biolysis of tissue, that the normal or abnormal manifestations of function, that the principles, or mechanical, electrical or catalytic effects, to be derived from inorganic matter in any of its infinite varieties of presentation, in either the body to be treated, or in that from which the substances or effects to be employed are derived, whether founded upon fancy or fact, hypothesis or demonstration, empiricism or science, has the slightest bearing upon the history, morbid anatomy, ætiology, diagnosis, prognosis, or treatment of a pathological condition is to prove the position of the essayist entirely within the bounds of his subject.

As it would be an absolute impossibility on an occasion like the present to fully consider all which the term therapeutics embraces, and being absolutely without advices from the chairman of our committee as to what portion of the subject would constitute his theme, or that of the other member of this committee, I have deemed it best to devote my attention to such general survey of the field, that my paper may serve as a preface to whatever the other gentlemen might choose to write.

To enter upon a proper consideration of my subject without further digression, I would say: A wise and gentle therapia would demand from the medical practitioner thorough investigation and consideration of each case. From a carefully ascertained previous history, (due allowance being made for the veracity of the narrator, in connection with his circumstances and surroundings, and the peculiar malady under which he may be laboring), deliberate and unbiased inspection, aided by the various appliances of modern diagnostic investigation, from palpation and percussion, to the thermometer, stethoscope and sphygmograph, he will be enabled to form a fairly accurate diagnosis and prognosis of his case. The therapeutical management thereof will then be governed in great measure by the empirical or (to say it more softly), clinical experience of himself, his teachers and confreres. Here, however, thanks to the modern system of recording cases, together with the light afforded by modern physiological research, in experimentation upon the lower orders of organized beings, as likewise to the flood of illumination which has been shed upon what was purely conjectural and occult, by that wonderful little instrument the microscope, he can act with some degree of scientific ability, even in cases which may be new to him; and he should never lose sight of the fact that the ætiology of a diseased condition gives the best indications for treatment; nor of that other fact, which can not be too often enforced upon the attention of the busy practitioner, that nature unaided is frequently the best physician; and that the duty of the medical attendant is oftener to simply remove the more prominent obstructions to function (by which I would indicate pain, pyrexia, œdma, dyspnœa, failure of heart power, cyanosis, or other urgent symptoms threatening immediate dissolution), and then patiently to watch and wait, in order that the natural forces may operate, than to write prescriptions or to administer medicines. To do nothing is frequently the best therapeutics!

The most manifest indication in the application of the therapeutical measures is upon the principle of counteracting starvation. The oft quoted passage, "Man cannot live by bread alone," is ever exemplified in the treatment of pathological condition; impaired function in any organ, or congeries of organs, induces direct starvation of certain of the fluids or tissues necessary to physiological continuity. Hope, faith, serenity of mind, cleanliness, disinfection, quiet, time, rest, sleep, in short, any and every measure promotive of assimilation and normal tissue-metamorphosis, are most potent therapeutical measures.

Promotion of elimination and excretion is the first step towards resumption of the normal processes of assimilation and nutrition. Not only must the cells composing the various glands and tissues, when they have fully completed their developmental condition, be cast off in order that the assimilative processes may be continued by their successors, but their contents must be liberated by disintegration of the cell walls, in order that each specific product may functionate. Again, the necessity for the secretion of products of these various glands and tissues is not greater than the necessity for excretion of their various components from the general circulatory current. And herein lies the most wonderful exhibition of the economy manifested in the ordinary processes of nature; in that those products of histolytic change, which, remaining in excess with the circulation, would result in speedy dissolution of the organism, excreted by the various emunctories of the body at the proper time and place, subserve repeatedly the purposes of the system, in the facial removal of egesta, and in the preparation of ingesta for assimilation and consequent nutrition. To this end, water (which is eliminated by so many channels of the body—the skin, the lungs, the kidneys, the bowels,) is usually the first, last, and greatest of therapeutic agencies. True, even water may be the chief factor in pathological states (as in asphyxia, by submersion, or in œdema under its various appellations of anasarca, hydrocephalus, ascites, etc., according to the locality overburdened therewith; states readily induced by imprudent exposure, or acute congestion of the kidneys, lungs, etc., whereby, instead of normal elimination, we may have extensive storage of fluids in the areolar tissue), but these are not diseases, in themselves considered, but only unpleasant manifestations of deprivation of oxygen, or inadequacy of functional activity when portions of the organisms are overtaxed; and in the majority of œdematous difficulties, the norm would most readily obtain, by administration of additional water, *iced*, while the patient was undergoing that most delightful therapeutic measure, the Turkish bath. As an adjuvant to other remedies, in many instances *aqua ad libitum* makes all the difference between success and failure in therapeutic practice.

Fluidity is the condition under which the action of the various vital currents remains a possibility; and the absorption and excretion of water is a principal means for the removal of histolytic products. Many so called diseases, the direct consequence of an excess of nitrogenous waste, are not diseases *per se*, but simlpy overaction of certain organs in the endeavor to maintain the integrity of the system—vicarious action. The plain indication in such cases is, First, to reduce the consumption of nitrogenous food; Second, to give large draughts of fluids (in order to assist the natural processes, by dissolving and washing out the accumulated salts); Third, to do nothing to prevent excretion from glands acting vicariously—which is simply nature's mode of cure—excepting by deobstruent efforts which shall restore the excretions to their proper channels, and by restoration of systemic tonicity.

Feb 2

In very many instances, tonics, so-called, do not prove to be tonic in the least when administered pure and simple, but combined with specifics counteracting deleterious material within the circulation, their effect is very soon beneficially apparent. We are rapidly returning to a consideration of the propriety of the ancient empirical practice of purgation and diuresis, as a preparation for treatment; this being one of the easiest and quickest methods of spurring the clogged glands to renewed effort, and unloading the circulation of material which has now become *debris.* But the subsequent therapeutical effort has very greatly changed for the better; and the skin is now one of the first of the emunctories to which remedial measures are addressed.

As this subject opens before me with a limitless and ever vanishing horizon, and as I have, doubtless, already exceeded the prescribed limit of time set apart for each report, I will perorate as follows:

Habits of cleanliness, good ventilation, abundant supply of good food, properly prepared, imbibition of a goodly quantity of fluids, orderly and timely attention to the various demands of the system, which necessarily implies regulation in both time and quantity of food and exercise taken, as also of the alvine and urinary dejections, and full satisfaction of the systemic demand for sleep, (taken at proper intervals), a persistent cultivation of that love for the neighbor which is a mainspring of a contented mind, together with an intensity of application to some regular pursuit which shall leave no time for ailments, are measures both prophylactic and therapeutic, which cannot fail with most persons, and to which the dentist is not a special exception, of a result which might *a priori* be predicted: the greatest measure of earthly happiness.

